# The Influence of Specimen Geometry and Loading Conditions on the Mechanical Properties of Porous Brittle Media

**DOI:** 10.3390/ma14237144

**Published:** 2021-11-24

**Authors:** Anatoly M. Bragov, Andrey K. Lomunov, Leonid A. Igumnov, Aleksandr A. Belov, Victor A. Eremeyev

**Affiliations:** 1 Research Institute for Mechanics, National Research Lobachevsky State University of Nizhny Novgorod, 603950 Nizhny Novgorod, Russia; lomunov@mech.unn.ru (A.K.L.); igumnov@mech.unn.ru (L.A.I.); belov_a2@mech.unn.ru (A.A.B.); 2 Department of Mechanics of Materials and Structures, Faculty of Civil and Environmental Engineering, Gdansk University of Technology, 11/12 Gabriela Narutowicza Street, 80-233 Gdańsk, Poland; eremeyev.victor@gmail.com; 3 Department of Civil and Environmental Engineering and Architecture (DICAAR), University of Cagliari, Via Marengo 2, 09123 Cagliari, Italy

**Keywords:** zirconia ceramics, brittle medium, porosity, Kolsky method, compressibility, strength, fracture, stress growth rate

## Abstract

Dynamic tests of fine-grained fired dioxide-zirconia ceramics under compression under uniaxial stress conditions were carried out. The influence of the specimen length on the obtained strength and deformation properties of ceramics is investigated. The thickness of the specimen has a significant impact on the course of the obtained dynamic stress–strain diagrams: short specimens have a much more sloping area of active loading branch. The main contribution to the modulus of the load branch resulting from tests of brittle porous media is made by the geometry of the specimens and the porosity of the material. When choosing the length of specimens for dynamic tests, the optimal geometry of the tested specimens is preferable in accordance with the Davies–Hunter criterion, when the contributions of axial and radial inertia are mutually compensated, and the contribution of the effects of friction in the resulting diagram is minimal. When choosing the geometry of specimens of brittle porous media, the structure of the material should be taken into account so that the size of the specimen (both length and diameter) exceeds the size of the internal fractions of the material by at least five times.

## 1. Introduction

For many years, intensive work has been carried out all over the world to study the dynamic properties of structural materials. Brittle porous materials have a good ability to damp shock and explosive effects. So, they are widely used in various designs of new technology. Porous materials play an important role as shock-damping material in containers for air, automobile, and other transportation of radioactive or highly toxic materials. Porous materials significantly reduce the load on the main protective structural elements in the event of emergency situations or terrorist attacks, accompanied by shock or explosive influences and high temperatures. To reliably determine the behavior of containers with concordant damping materials under impact data on their properties are required, mainly, dynamic stress–strain curves. In addition, layered structures for protection against damaging effects by bullet and fragmentation elements are a possible field of application for porous low-density materials. In these designs, the gap between the metal layers is filled with a light material with good damping, dissipative and thermophysical properties.

The compressibility of metals under impact have been studied at present in more detail than the properties of brittle materials, such as concretes, ceramics, and refractories, which are associated with additional requirements imposed on test installations when studying this class of materials. Currently, there are no generally accepted standards for installations for the study of brittle and especially structurally inhomogeneous materials. A large number of types of various materials fall under the name of brittle and structurally heterogeneous, these are concretes, rocks, ceramics, various types of frozen materials (ice, bitumen, frozen soils, etc.). In their chemical and structural composition, all these materials in the field of dynamic loading are still insufficiently studied, so obtaining new data on their properties remains relevant.

The study of the mechanical properties of ceramic materials even under quasistatic effects is a very difficult and time-consuming task, since their properties depend on a large number of factors (multicomponent, porosity, humidity, etc.). Methodological difficulties in the study of the mechanical properties of ceramics and concrete repeatedly increase under dynamic loads, characterized by high intensities and short exposure times.

Brittle materials, in contrast to ductile ones, have a small deformation of destruction (often no more than 1%), and therefore, if the loading takes place too quickly, as in the usual SHPB test [1], the specimen can begin to break unevenly, i.e., the front of the specimen may be destroyed while the back remains intact. The conditions for the constancy of the strain rate and the equilibrium of stresses at the ends of the specimen should be satisfied during most of the test. A non-dispersive smoothly increasing pulse in the loading bar is required to test brittle materials (ceramics, rocks), which have an almost linear dependence of the stress–strain curve up to fracture. If the incident pulse is formed with a steep leading edge, then it is not possible to achieve dynamic stress equilibrium in the sample of a brittle material, and the specimen can immediately collapse at its end in contact with the loading bar after the arrival of the incident wave [2,3].

For SHPB test measurements to be valid, the dynamic load must increase slowly enough that the specimen is subjected to an almost quasi-static load; in this case, the deformation of the specimen will be uniform. The possibility of using the incident pulse shaper in the SHPB system was considered in [4]. It was noted that a smoothly rising incident pulse is preferred to minimize the dispersion and inertia effects and so to contribute to the dynamic equilibrium of the specimen stress state.

The easiest and most convenient way to create an incident pulse with an inclined front is to attach a small thin disk of soft material to the impact end of the loading pressure bar [4,5]. Such a disk, called a pulse shaper, can be made of copper, aluminum, brass or rubber with a thickness of 0.1–2.0 mm.

When studying the behavior of several ceramics under pulsed loading, it was noted [6] that the thickness of the specimen significantly affects the course of the obtained dynamic stress–strain curves. Specimens with a diameter of 20 mm and with a thickness from 2 mm to 10 mm were studied. It was determined that thicker specimens have a greater value of the modulus of the load branch and less energy absorption. Strength properties depend weakly on the length of the specimen. A similar trend was also noted in [7] when studying specimens of fine-grained concrete of 10 mm and 20 mm length: thicker specimens have a larger modulus of the load branch, while the breaking stress is practically independent of the length of the specimen.

The aim of this work was to evaluate the influence of the geometry of specimens of a brittle porous medium using the example of fine-grained firing ceramics based on zirconia on the deformation characteristics under compression at uniaxial stress condition.

## 2. Experimental Method

**Figure 1:**
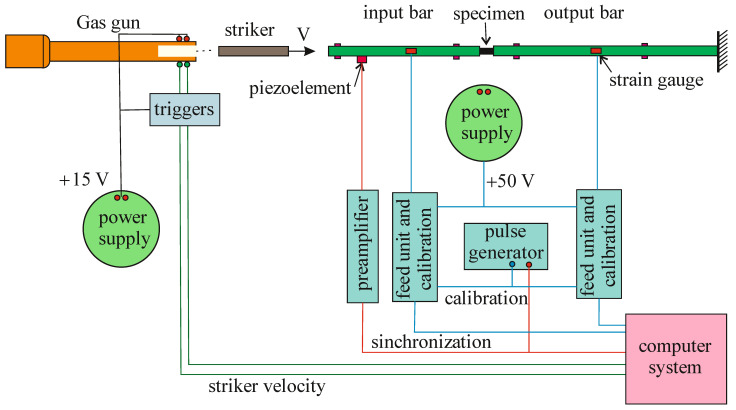
Experimental setup scheme.

We used the original setup [5] that implements the Kolsky method [1] with a split Hopkinson pressure bar (SHPB) for investigation the dynamic properties of ceramics under compression. The experimental stand consists of a gas gun with a control system and a complex of measuring and recording equipment. Pressure bars with a diameter of 20 mm from D16T alloy are equipped with low-base strain gauges (Figure 1). The amplitude of the incident pulse, proportional to the striker velocity, varied from 60 MPa to 180 MPa, respectively, the strain rate (taking into account the different lengths of the specimens) was from 350 s^−1^ to 6000 s^−1^.

In the test, a one-dimensional elastic loading (incident) impulse ε*^i^*(*t*) of the required amplitude and duration (determined by the speed and length of the striker), propagating along the incident bar with the speed of sound *C*, reaches the specimen and loads it; while part of the wave is reflected back by the reflected pulse ε*^r^*(*t*), and part passes into the supported bar by the transmitted pulse ε*^t^*(*t*). Based on these strain pulses recorded by strain gauges in measuring bars, the parametric dependences of the development of the axial stress σ*_s_*(*t*), strain ε*_s_*(*t*) and strain rate ${\dot{\varepsilon }}_{s}(t)$ components of the specimen over time by the Kolsky method formulas [8,9] were determined:

(1) $$\sigma_{s}(t)=\frac{EA}{A_{S}^{0}}\varepsilon^{T}(t)$$

(2) $$\varepsilon_{s}(t)=-\frac{2C}{L_{0}}\int_{0}^{t}\varepsilon^{R}(t)\cdot dt$$

(3) $${\dot{\varepsilon }}_{s}(t)=-\frac{2C}{L_{0}}\cdot \varepsilon^{R}(t)$$

where *E* and *A* are Young’s modulus and cross-sectional area of the output bar, *C* is the speed of elastic waves in the input bar, *L*_0_ is the initial length of the specimen, $A_{s}^{0}$ is the specimen initial cross-sectional area.

Then, after synchronizing the initial pulses, we can construct a dynamic diagram σ_s_(ε_s_) with a dependence ${\dot{\varepsilon }}_{s}(\varepsilon_{s})$.

When constructing dynamic stress–strain curves by the Kolsky method, sufficiently accurate matching in time of strain pulses is required, including expert selection of the starting point of reference for each pulse. A characteristic feature of the tests of porous brittle media by the Kolsky method is the presence of a significant (up to 20 μs) delay in the transmitted pulse relative to the reflected one, even in the case of gluing of both recording strain gauges at the same distance from the specimen. This is due to several reasons:

- Considerable difference in the acoustic impedances of the sample material and the material of the pressure bars;
- Low speed of wave propagation in a porous medium;
- The quality of the processing of the end surfaces of the specimen;
- Significant porosity of the material.

In addition, the registration of weak signals from strain gauges is sometimes accompanied by electromagnetic interference superimposed on the zero line. These factors lead to the fact that there is a probability of error when choosing the start points of these pulses.

The procedure of pulse synchronization is as follows. The beginning point of the incident pulse is the time point after which the first deflection of the recorded ray from the zero line is observed. Further, the positions of the starting points of the reflected and transmitted pulses are determined using the known elastic wave speed in the measuring bars and the known distance of the strain gauges from the specimen. When the sensors are glued at the same distance from the specimen, these points are selected synchronously.

At given starting points, it is checked the execution of the main condition of the Kolsky method, namely, the strains uniformity in the specimen body by checking the equality of deformations at the ends of the specimen during the test:

ε*^i^*(*t*) + ε*^r^*(*t*) = ε*^t^*(*t*) (4)

The original synchronization program allows one to automatically select and manually adjust the relative position of these pulses, but for most tests, this adjustment is not required. In this procedure, the main attention is paid to the fulfillment of condition (4) with the smallest error over the entire pulse duration, with the exception of the initial section (several microseconds), during which the stress–strain state of the specimen cannot be considered as uniform. Studies have shown that when testing concretes, ceramics, and some other porous brittle media, the assumption of equal forces at the ends of the specimen is quite good.

Elastic strain impulses in the pressure bars are measured using small-base foil strain gauges, then stored by a multi-channel digital oscilloscope and transmitted to a personal computer for processing and analysis. The original processing program allows one to synchronize the selected pulses and build true stress–strain diagrams. If necessary, a controlled smoothing of recorded pulses using integral splines is possible. It is also possible to conduct statistical and regression analyses of the results.

Because of the large contrast in the acoustic impedances *ρ*C of the input pressure bar and the specimen of the porous material, the reflected pulse amplitude can reach 80–90% of the amplitude of the incident wave. So, the specimen will be exposed to several loading cycles. In order to authentically register a repeated loading cycles during one experiment, it is necessary to exclude the influence on the loading process in the second and subsequent cycles of the transmitted pulse reflected from the rear end of the transmitting bar. For that, the length of the transmitting bar should be increased in comparison with the length of the incident bar [10,11]. In this series of experiments, the length of incident and transmitting bars were 1.5 m and 4.5 m, respectively. As a result, it possible to register the main and two additional loading cycles.

## 3. Tested Specimens

There were tested specimens of zirconia ceramics, a promising material for use in the nuclear industry. From a chemical point of view, this material is relatively inert, does not form fusible compounds with uranium dioxide, and its melting point is about 3000 K. Materials based on zirconium dioxide have a thermal conductivity 1.5–2 times lower than materials based on other highly refractory oxides.

The raw material for the manufacture of ceramic specimens was a material obtained from cubic zirconia stabilized with yttrium oxide Y_2_O_3_ in a molar fraction of 11–12%. Manufacturing technology was close to factory. To obtain the required fractional composition of ceramics (Table 1), the technology included grinding, chemical and magnetic cleaning, the addition of a temporary plasticizing binder, pressing (pressure 100 MPa) and annealing (2000 K for 13 h).

These ceramics are characterized by the following physicomechanical properties under normal conditions: density 4.7–4.9 g/cm^3^, porosity 20%, static compressive strength 39 MPa.

Specimens for testing were made in the form of a cylinder with a diameter of ~20 mm and a thickness of ~10 mm. This geometry of the specimens was chosen in accordance with the Davies—Hunter recommendation [12] to minimize the effects of inertia and friction. The ideal specimen slenderness ratio (that is, the ratio of its length to diameter) has been studied for a long time, as it plays an important role in inertial effects during dynamic SHPB testing. Based on a joint analysis of the effects of axial and radial inertia, Davies and Hunter [12] proposed an optimal ratio of sample slenderness *L*/*D* = √3·*ν*_s_ /2; where *L* and *D* are the length and diameter of the cylindrical specimen, respectively, and *v_s_* is the Poisson’s ratio of the material under test. With this *L*/*D* ratio, the components of axial and radial inertia are mutually compensated, therefore, the calculated stress in the sample is considered reliable. This ratio is valid for a variety of materials, including brittle media.

In addition, to assess the influence of the geometry of the specimens on the resulting stress–strain diagrams, some of the specimens were made with thicknesses of 5 mm and 2 mm.

One of the characteristic features of the deformation and fracture of brittle materials (and ceramics just refer to such materials) is a significant effect on the deformation and strength characteristics of the state of the surface of the specimens. The presence of micro- or macrocracks on the surface, barbs and cavities leads to a significant decrease in the strength properties of brittle materials. Therefore, the specimens before testing were subjected to manual grinding on sandpaper with a grain size of 0.01 mm. When establishing the specimen in the working position, to reduce friction and improve the acoustic contact between the ends of the measuring bars and the specimen, there were 2–3 layers of a thin (10 μm) fluoroplastic film.

Ceramic specimens were tested under compression conditions of a one-dimensional stress state. The specimen temperature in all experiments was 20 ± 2 °C.

## 4. Results and Discussion

Ceramic specimens were tested for compression using a device that implements the Kolsky method. During testing, by varying the striker velocity (i.e., the amplitude of the incident pulse), loading modes were selected in which the specimen after test either retained its apparent integrity and strength, or collapsed. Visual control of the samples after the experiments made it possible to assess the sample destruction degree. However, such an examination does not give an unambiguous answer to the question at what point the specimen collapsed. It is known that for tested materials with a low acoustic impedance *ρC*, the reflected pulse can have significant amplitude. This impulse, after reaching the impacted end-face of the incident bar, reflects from it as a compression wave and, after reaching the specimen, reloads it, then again partially reflects and so on. Such a process is repeated many times until this pulse is completely faded away. As a result of repeated loading cycles, the resulting microfractures have the ability to develop and enlarge. In addition, unloading waves from the free side surface of the specimen can affect the fracture process. However, the choice of the optimal specimen slenderness due to the Davies–Hunter recommendation [12], as well as the analysis of pulses in the bars, give reason to believe that there is no such effect.

**Figure 2:**
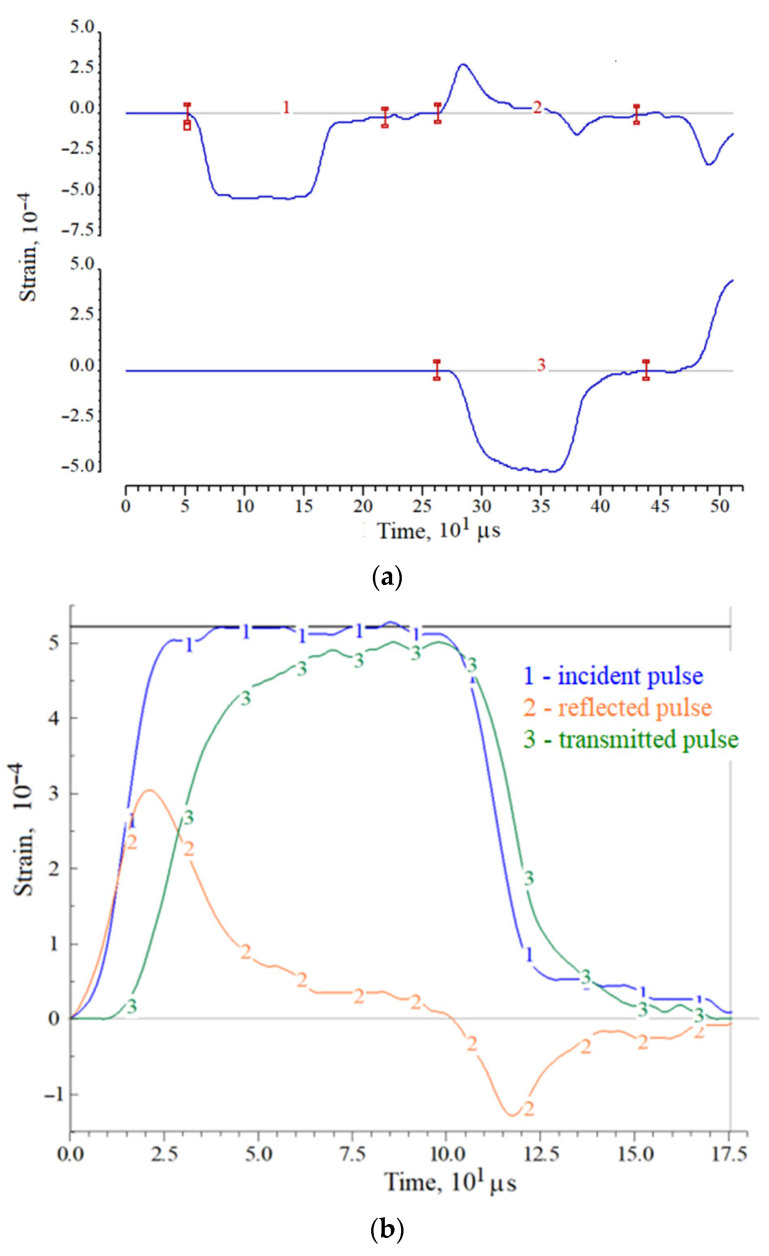
An example of strain pulses in measuring bars during registration (**a**) and during synchronization (**b**) when testing a ceramic specimen maintaining its visible integrity.

**Figure 3:**
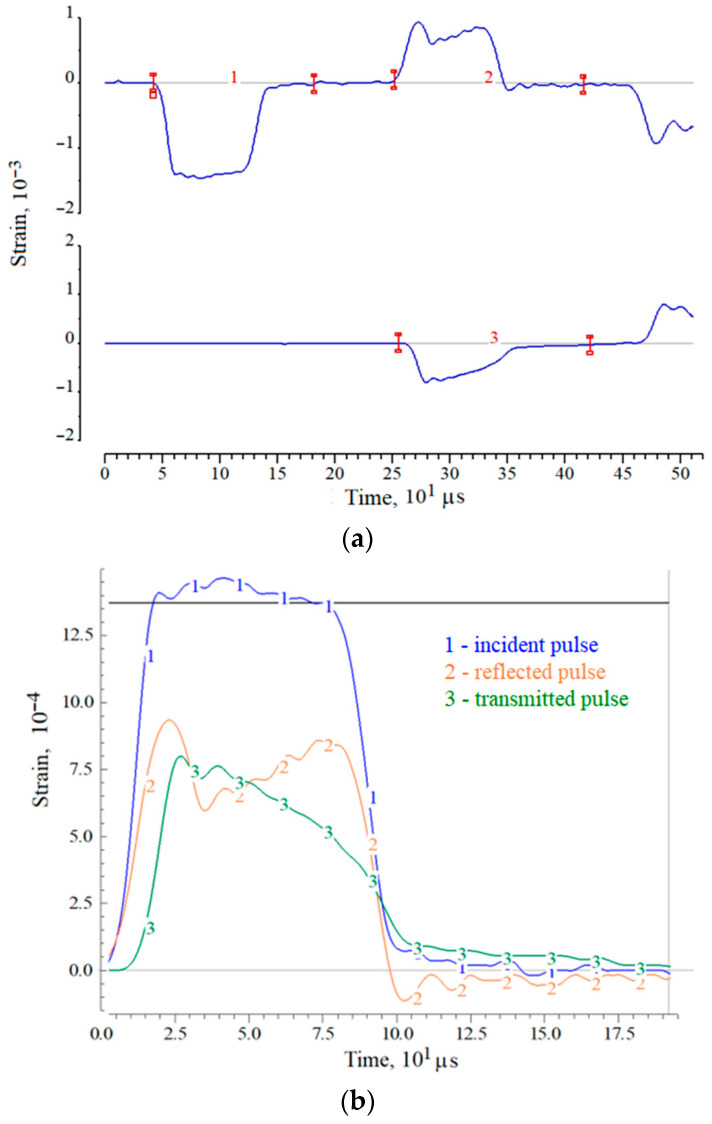
An example of strain pulses in measuring bars during registration (**a**) and during synchronization (**b**) when testing a ceramic specimen with its complete destruction.

A more accurate answer about the destruction of specimen in the first loading cycle may be given after the analysis of deformation pulses recorded in the pressure bars. As an example, Figure 2 and Figure 3 show the initial pulses in the measuring bars, both during registration (a) and in the process of pulses synchronization (b). Moreover, the pulses in Figure 2 correspond to the case of maintaining the apparent integrity of the specimen, and the pulses in Figure 3 correspond to the case of complete destruction of the specimen (into powder). In these figures, the initial pulses recorded in the pressure bars are shown: 1—incident pulse, 2—reflected pulse and 3—transmitted pulse.

In the case of absence of the sample destruction under loading by a trapezoidal incident pulse ε_i_(*t*) with a flat top, the reflected pulse ε_r_(*t*) (the sample strain rate) first increases and then decreases due to an increase in the resistance of the sample during its deformation. After the end of affecting of the incident pulse on the sample, the transmitted pulse ε_t_(*t*) also begins to decrease, while the strain rate determined by the reflected pulse ε_r_(*t*) becomes negative. Thus, in the section of the sample active loading, both stress and deformation increase, then, with the beginning of the incident pulse decrease, the stress in the specimen decreases to almost zero, while the achieved deformation decreases by a certain amount, determined by the unloading capacity of the material and it is calculated by using the negative part of the reflected pulse.

A different picture occurs in the case of destruction of the specimen. A simultaneous increase in stress (pulse ε_t_(*t*)) and strain rate (pulse ε_r_(*t*)) takes place at the initial stage of specimen loading, however, after the point of maximum stress, the avalanche-like fracture process begins in the specimen. The stress after this point decreases, whereas the strain rate increases. So, although the amplitude of the incident pulse remains almost constant, the collapsing specimen does not completely pass the compression wave, its resistance to deformation is steadily decreasing.

**Figure 4:**
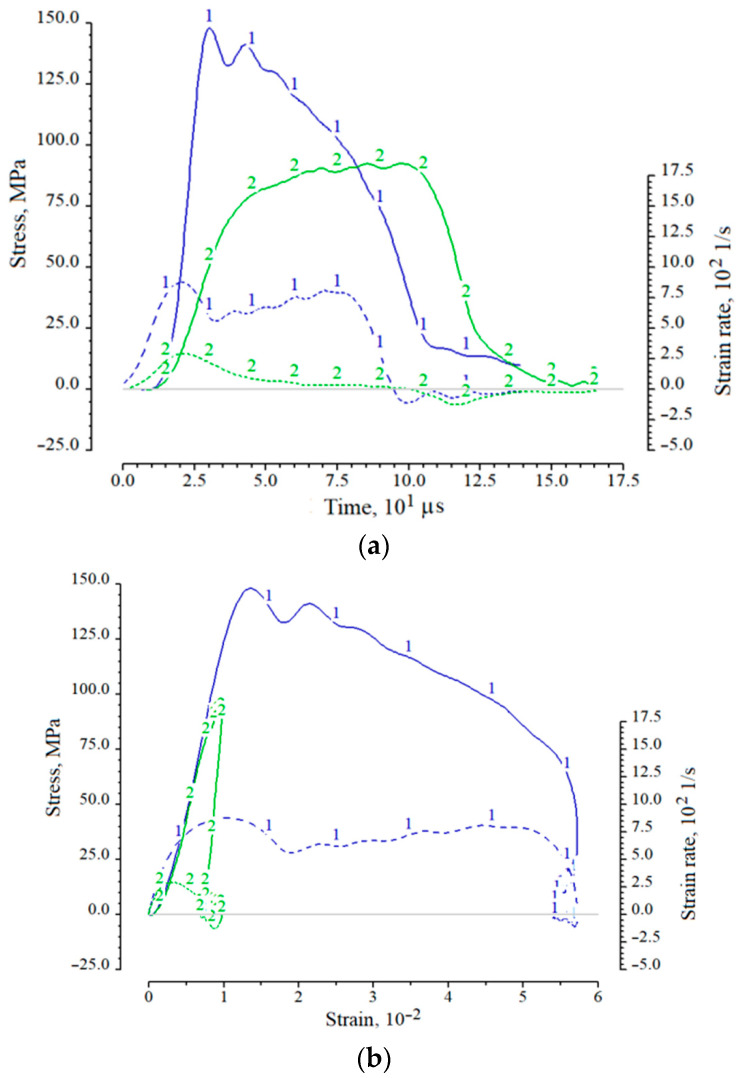
Examples of functions σ*_s_*(*t*) and ${\dot{\varepsilon }}_{s}(t)$ (**a**) and σ*_s_*(ε*_s_*) and ${\dot{\varepsilon }}_{s}(\varepsilon_{s})$ (**b**) for cases of maintaining integrity (curves 2) and complete destruction of a specimen (curves 1).

Examples of resulting charts corresponding to these two types of tests are presented in Figure 4 in the form of parametric dependences σ*_s_*(*t*) and ${\dot{\varepsilon }}_{s}(t)$, as well as the diagrams σ*_s_*(ε*_s_*) and ${\dot{\varepsilon }}_{s}(\varepsilon_{s})$ themselves. The functions ${\dot{\varepsilon }}_{s}(t)$ and ${\dot{\varepsilon }}_{s}(\varepsilon_{s})$ are represented on the graphs by dotted lines in the lower half of the figure field. The corresponding axis is located on the right side of the graphs. Digital markers on the lines are used to identify the curves and their mutual reference.

For both cases, one can note the nonlinearity of the initial section of the load branch and the significant difference between the load and unload branches. In the case of fracture of the specimen, the stress after reaching a maximum value begins to decrease with a constant increase in strain. Due to the short duration of the deformation process, the destroyed specimen particles that are not connected to each other during the test remain between the ends of the measuring rods, and their partial compaction occurs. This process is similar to high-speed deformation of non-cohesive soils. After the end of the incident pulse, the compacted ceramic particles have a small unloading capacity, which reveals in the form of a section of the diagram with partial restoration of the sample shape (negative portion of the function ${\dot{\varepsilon }}_{s}(\varepsilon_{s})$).

**Figure 5:**
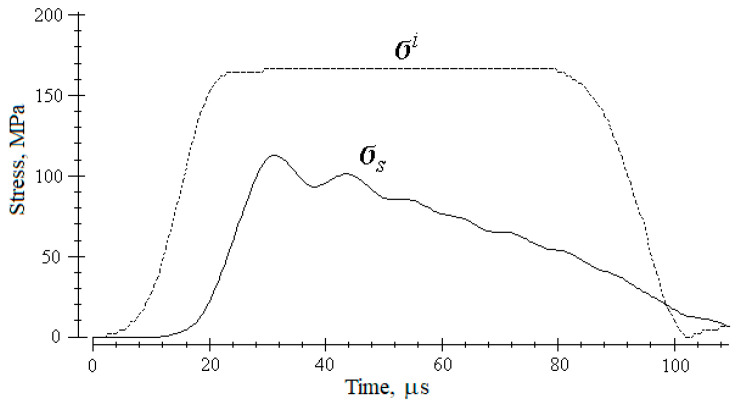
The parametric process of stress development in a specimen σ*_s_* in case of its destruction in comparison with a loading pulse σ*^i^*.

When the specimen is loaded with a high-intensity pulse, the destruction of the specimen occurs directly in the load wave; the proof of that can be illustrated in Figure 5, where the parametric processes σ*_i_*(*t*) and σ*_s_*(*t*) are shown.

**Figure 6:**
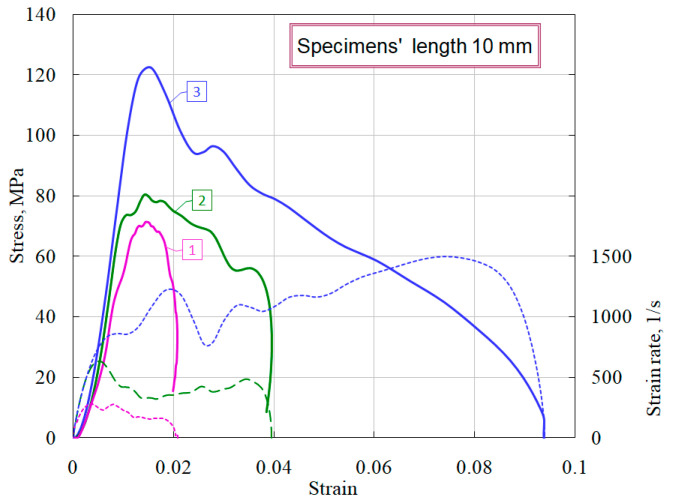
Deformation diagrams of ceramic specimens of optimal geometry under compression.

The results of compression tests under uniaxial stress state of ceramic specimens of optimal geometry (diameter 20 mm, length 10 mm) are presented in Figure 6.

The solid lines in the figure show the characteristic diagrams of dynamic deformation, which are the result of averaging several experiments conducted under nominally identical conditions. The dashed lines of the corresponding colors at the bottom of the figure show the history of changes in the strain rate of the specimen. Curve 1 corresponds to the conditions under which, after the experiment, the specimen retained its visible integrity. Curve 3 was obtained under conditions of complete destruction of the specimen. Curve 2 was obtained under conditions when the specimens either had small (mainly peripheral) fractures, or, retaining the whole appearance, lost their structural connectivity.

It is clearly seen that the structural strength of ceramics is about 70 MPa, however, during the dynamic loading, higher stress values were obtained, as evidenced by the upper curves 2 and 3. This behavior of the materials is due to the dynamic nature of the load and is determined by two competing processes occurring in specimen: the process of formation, growth and fusion of microcracks and micropores into macropores and cracks, on the one hand, and the wave nature of the increase of load in the material, on the other. It should be noted that the sample clamped between the ends of the measuring bars, due to the inertia of the process of its deformation, even when the process of internal damage has begun, remains in place and continues to transmit through itself a compressive stress wave of increasing amplitude. So, if the rate of increase of stress exceeds the velocity of the fracture process, then the specimen with already formed and developing fracture centers can be overloaded, i.e., it can withstand for some time ever-increasing loads. Similar phenomena were discussed in the analysis of the temporal dependence of compressive strength in [13,14].

**Figure 7:**
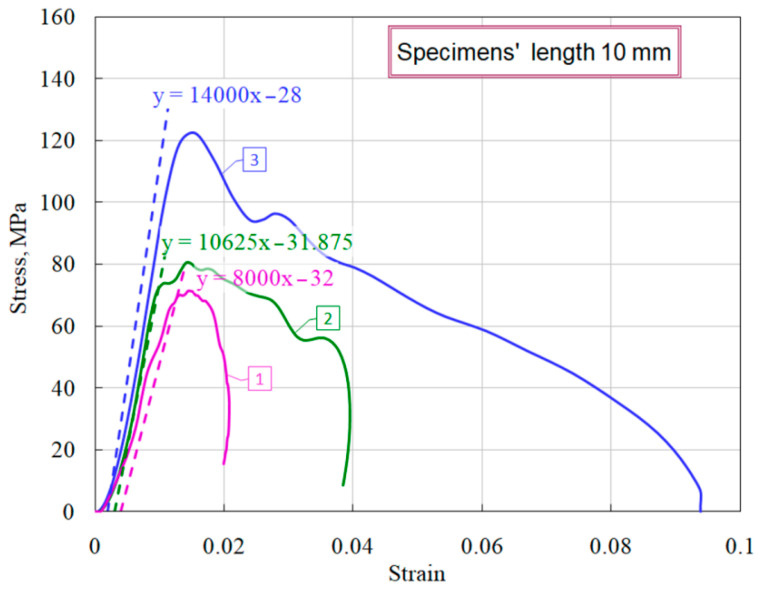
Definition of modules of load branches.

**Figure 8:**
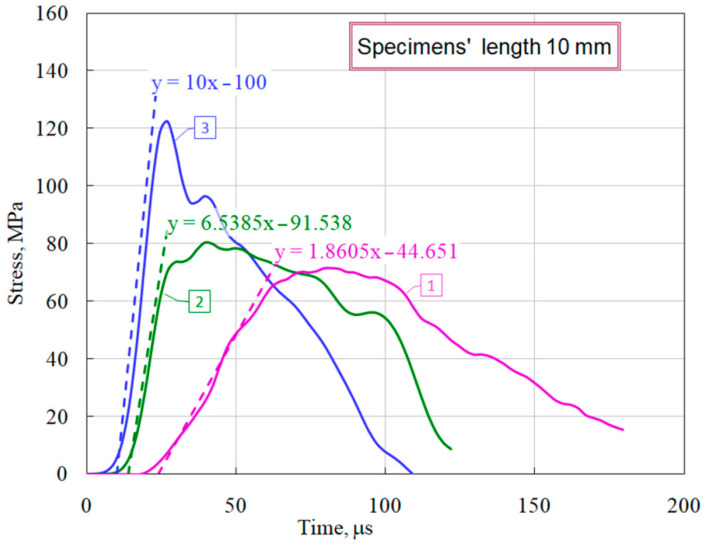
Definition the stress growth rate.

Using the obtained stress–strain charts, the average values of the modules of the load branches (*d*σ/*d*ε) were measured (Figure 7), as well as there were determined the maximum stresses that the specimen withstood before failure began at various levels of the strain rate and the corresponding times at the onset of fracture (the beginning of the decay of the σ*_s_*(*t*) curve. These parameters for each curve are given in Table 2. When processing the experimental information for each diagram, in addition to the average strain rate ${\dot{\varepsilon }}_{s}$ of the sample, we determined the maximum values of the stress growth rate ${\dot{\sigma }}_{s}$ in the sample (Figure 8), which are also shown in Table 2.

**Figure 9:**
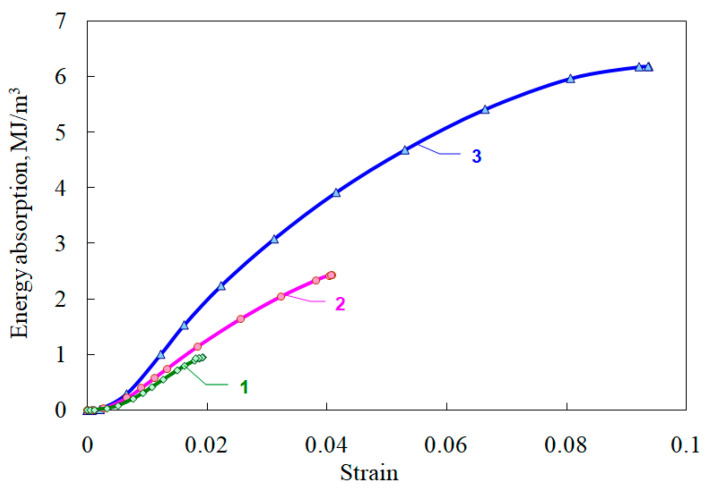
Energy absorption of ceramics.

An important characteristic of ceramics working as a protective (energy-absorbing) material is their energy capacity, calculated as the area under the curve σ~ε (Figure 9). This characteristic is also given in Table 2.

**Figure 10:**
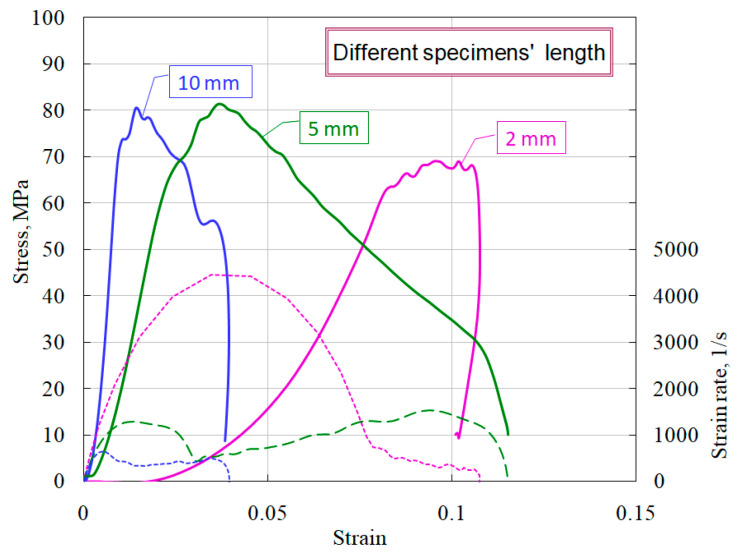
Effect of specimens’ length on ceramic properties.

As noted earlier [6,7], the thickness of the specimen has a significant effect on the course of the obtained dynamic stress–strain curves. In this regard, a comparative study of the influence of various geometries of ceramic specimens on mechanical characteristics was carried out. Figure 10 shows the average diagrams during compression of specimens with a diameter of 20 mm and a thickness of 10 mm, 5 mm, and 2 mm.

**Figure 11:**
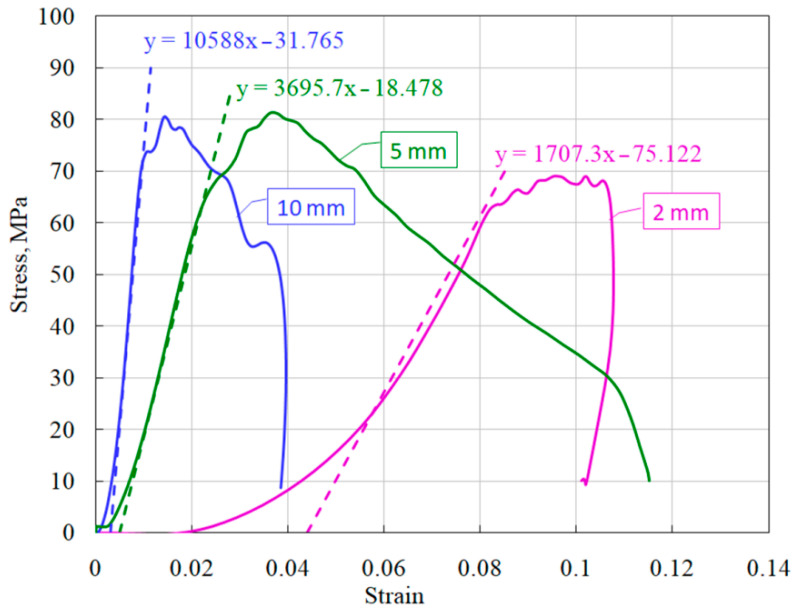
Definition of modules of load branches of ceramic diagrams for specimens of different lengths.

It is clearly seen that short specimens have a much more sloping area of active loading. Using the obtained stress–strain diagrams, the average values of the deformation module *d*σ/*d*ε (steepness of the active loading section) were measured (Figure 11). The dashed lines show the linear approximations of the sections of active loading with the corresponding equations.

A similar trend was noted earlier in the study of ceramics [6] and fine-grained concrete [7]. The reason for the greater deformability of specimens of brittle media of shorter length can be as follows. Specimens whose length is 5 times different have approximately the same porosity and grain size. In addition, they have similar roughness characteristics of the end surfaces and possible deviations in their parallelism. When the incident strain (stress) pulse reaches the end of the specimen, it is divided into reflected and transmitted. These two pulses are the responses of the material to the applied load. In accordance with the formulas of the Kolsky method (1)–(3), the strain rate is directly proportional to the reflected pulse, and the specimen deformation is proportional to the integral of the reflected pulse. The voltage in the specimen is directly proportional to the transmitted pulse. The partition of the fractions of the reflected and transmitted pulses in the incident pulse is determined by a number of factors. The magnitude of the reflected wave is determined primarily by the ratio of impedances *ρC* of the incident bar and the specimen. In addition, in general, the ratio of reflected and transmitted waves is determined by the mechanical rigidity of the specimen material as well as its porosity. However, at the initial moment of exposure of the incident pulse to the specimen, this ratio is also determined by the roughness of the surface of the specimen, the non-flatness of its end and the possible non-parallelism of the end surface of the incident bar and the loading end surface of the specimen. Naturally, the shorter the length of the specimen, the greater the contribution of these defects to the reflected pulse, which determines the deformation of the specimen at the initial stage of loading, and, accordingly, the less the deformation modulus of the loading branch of the stress–strain diagram. Since, in accordance with Equation (2), the deformation of the specimen is inversely proportional to its length, then, for the same amplitude of the reflected pulse, the deformation of the 2-mm long specimen will be five times larger than for the 10-mm long specimen 10. This fact determines the corresponding difference obtained in the deformation modules of the sections of active loading of specimens of various lengths.

However, it should be noted that specimens of tested ceramics with a length of 2 mm cannot be considered representative, since, as follows from the granulometric composition of the ceramic (Table 1), fractions of 0.63–1 mm in size are present in it, in an amount of 40 wt.%. When choosing the geometry of specimens of brittle porous media, the structure of the material should be taken into account so that the size of the specimen (both length and diameter) exceed the size of the internal fractions of the material by at least five times.

Thus, in dynamic tests of brittle porous media, the optimal geometry of the test specimens, determined due to the Davies–Hunter recommendation [12], is preferable when the contributions of axial and radial components of inertia are mutually compensated, and the contribution of the effects of friction to the resulting diagram is minimal.

## 5. Conclusions

In dynamic tests of brittle porous media, special attention should be paid to quality control of the end surfaces of the specimen: to ensure minimal roughness, as well as parallelism of the ends and their flatness. The thickness of the specimen has a significant effect on the course of the obtained dynamic stress–strain diagrams: short specimens have a much more sloping area of active loading. The main contribution to the modulus of the load branch resulting from tests of brittle porous media is made by the geometry of the specimens and the porosity of the material. When choosing the length of specimens for dynamic tests, the optimal geometry of the tested specimens, determined due to the Davies–Hunter recommendation [12], is preferable when the contributions of axial and radial components of inertia are mutually compensated, and the contribution of the effects of friction in the resulting diagram is minimal.

## Figures and Tables

**Table 1:** Granulometric composition of ceramics (wt.%).

Sizes of Fractions, mm					
<0.05	<0.2	0.2–0.315	0.315–0.4	0.63–1	1–2
40	-	20	-	40	-

**Table 2:** Results of dynamic tests of ceramics.

Curve Number in the Diagram	The Module of the Load Branch, MPa	Average Strain Rate, 1/s	Stress Growth Rate, MPa/μs	Destruction Start Point			
				Strength, MPa	Strain, %	Time, μs	Energy Capacity, MJ/m^3^
1	8000	220	1.9	71	1.5	82	0.93
2	10,625	400	6.5	80	1.4	40	2.42
3	14,000	1040	10.0	113	1.4	27	6.18

## Data Availability

Not applicable.
